# Platelet mitochondrial dysfunction in critically ill patients: comparison between sepsis and cardiogenic shock

**DOI:** 10.1186/s13054-015-0762-7

**Published:** 2015-02-11

**Authors:** Alessandro Protti, Francesco Fortunato, Andrea Artoni, Anna Lecchi, Giovanna Motta, Giovanni Mistraletti, Cristina Novembrino, Giacomo Pietro Comi, Luciano Gattinoni

**Affiliations:** U.O. Terapia Intensiva ‘Emma Vecla’, Fondazione IRCCS Ca’ Granda – Ospedale Maggiore Policlinico, Università degli Studi di Milano, via F.sco Sforza 35, 20100 Milan, Italy; U.O. Neurologia – Centro Dino Ferrari, Fondazione IRCCS Ca’ Granda – Ospedale Maggiore Policlinico, Università degli Studi di Milano, via F.sco Sforza 35, 20100, Milan, Italy; Centro Emofilia e Trombosi Angelo Bianchi Bonomi, Fondazione IRCCS Ca’ Granda – Ospedale Maggiore Policlinico, Università degli Studi di Milano, via F.sco Sforza 35, 20100, Milan, Italy; U.O. Anestesia e Rianimazione, A.O. San Paolo, Università degli Studi di Milano, via A. Di Rudinì 8, 20100, Milan, Italy; Laboratorio Centrale di Analisi Chimico Cliniche e Microbiologia, Fondazione IRCCS Ca’ Granda – Ospedale Maggiore Policlinico, Università degli Studi di Milano, via F.sco Sforza 35, 20100, Milan, Italy

## Abstract

**Introduction:**

Platelet mitochondrial respiratory chain enzymes (that produce energy) are variably inhibited during human sepsis. Whether these changes occur even during other acute critical illness or are associated with impaired platelet aggregation and secretion (that consume energy) is not known. The aims of this study were firstly to compare platelet mitochondrial respiratory chain enzymes activity between patients with sepsis and those with cardiogenic shock, and secondly to study the relationship between platelet mitochondrial respiratory chain enzymes activity and platelet responsiveness to (exogenous) agonists in patients with sepsis.

**Methods:**

This was a prospective, observational, case–control study. Platelets were isolated from venous blood of 16 patients with severe sepsis or septic shock (free from antiplatelet drugs) and 16 others with cardiogenic shock, within 48 hours from admission to Intensive Care. Platelet mitochondrial respiratory chain enzymes activity was measured with spectrophotometry and expressed relative to citrate synthase activity, a marker of mitochondrial density. Platelet aggregation and secretion in response to adenosine di-phosphate (ADP), collagen, U46619 and thrombin receptor activating peptide were measured with lumiaggregometry only in patients with sepsis. In total, 16 healthy volunteers acted as controls for both spectrophotometry and lumiaggregometry.

**Results:**

Platelets of patients with sepsis or cardiogenic shock similarly had lower mitochondrial nicotinamide adenine dinucleotide dehydrogenase (NADH) (*P* < 0.001), complex I (*P* = 0.006), complex I and III (*P* < 0.001) and complex IV (*P* < 0.001) activity than those of controls. Platelets of patients with sepsis were generally hypo-responsive to exogenous agonists, both in terms of maximal aggregation (*P* < 0.001) and secretion (*P* < 0.05). Lower mitochondrial NADH (*R*^*2*^ 0.36; *P* < 0.001), complex I (*R*^*2*^ 0.38; *P* < 0.001), complex I and III (*R*^*2*^ 0.27; *P* = 0.002) and complex IV (*R*^*2*^ 0.43; *P* < 0.001) activity was associated with lower first wave of aggregation with ADP.

**Conclusions:**

Several platelet mitochondrial respiratory chain enzymes are similarly inhibited during human sepsis and cardiogenic shock. In patients with sepsis, mitochondrial dysfunction is associated with general platelet hypo-responsiveness to exogenous agonists.

**Trial registration:**

ClinicalTrials.gov NCT00541827. Registered 8 October 2007.

## Introduction

The pathogenesis of multiple organ failure during sepsis remains unclear. According to one hypothesis, overt systemic inflammation in response to infection inhibits mitochondria (the ‘powerhouse of the cell’ [1]). As a consequence, organs become unable to produce enough energy to maintain their normal activities: the organs enter into a hypometabolic state and lose their functions [2,3].

Testing this hypothesis in humans is problematic because of difficult access to vital organs. Circulating platelets are rich in mitochondria and can be obtained easily even from critically ill patients. Platelet mitochondrial function can be assessed with different techniques. For instance, spectrophotometry can measure the activity of enzymes that form the respiratory chain and are devoted to energy production [4] in platelets just as in other tissues [5,6]. Using spectrophotometry, we [7] and other authors [8] have noted that these mitochondrial enzymes are variably inhibited in platelets of septic patients. However, no one has ever clarified whether these changes are specific to sepsis (in order to better define underlying mechanisms) or whether they are related to changes in platelet aggregation and secretion in response to agonists (to verify the association between mitochondrial inhibition and cellular loss of function).

In resting platelets, mitochondrial respiration normally accounts for three-quarters of energy production, with glycolysis providing the rest [9]. Metabolic ATP and ADP are in the cytoplasm with a ratio of 7 to 10 and represent one-third of total intraplatelet adenine nucleotides. Nonmetabolic ATP and ADP are segregated into dense (δ) granules with a ratio of around 1 and represent the other two-thirds of total adenine nucleotides; they are secreted upon cellular stimulation and are essential for the late phase (or second wave) of aggregation [10]. Therefore, most of the total intraplatelet ATP is in the cytoplasm (metabolic pool) whereas most of the total ADP is in δ-granules (storage pool). Serotonin is another constituent of δ-granules. Following platelet activation, mitochondrial respiration and glycolysis accelerate to produce extra metabolic ATP to sustain shape change, aggregation and secretion [11,12]. This incremental energy consumption is one major determinant of platelet function [12]. When mitochondria are inhibited, glycolysis alone can produce enough energy for resting cells, but not for activated cells. This is the reason why mitochondrial inhibitors diminish platelet inducible (and energy demanding [13]) responses [14-18].

The aims of this study were to compare platelet mitochondrial respiratory chain enzyme activity between patients with sepsis and those with cardiogenic shock (when the trigger for inflammation is hypoperfusion and not infection) and to study the relationship between mitochondrial respiratory chain enzyme activity and platelet responsiveness to (exogenous) agonists in patients with sepsis.

## Materials and methods

This study was approved by the Ethics Committee of the Fondazione IRCCS Ca’ Granda – Ospedale Maggiore Policlinico, Milan, Italy and was registered on 8 October 2007 at ClinicalTrials.gov (NCT00541827). Written consent was obtained from patients (when they returned conscious) and healthy volunteers.

We enrolled 16 adults with severe sepsis or septic shock [19] and 16 others with cardiogenic shock [20] within 48 hours from admission to intensive care. Sixteen healthy volunteers matched for sex and age acted as controls. Exclusion criteria were platelet transfusion within the last 15 days, severe thrombocytopenia (<20 × 10^3^ platelets/mm^3^) or anaemia (<8 g/dl) and known mitochondrial disease. In addition, patients with sepsis and controls were excluded if they had received anti-serotoninergic or anti-platelet drugs within the last 15 days. This criterion did not apply to patients with cardiogenic shock, who were expected to have received anti-platelet drugs (for acute coronary syndrome) by the time they were admitted to intensive care. We therefore decided *a priori* to study mitochondrial respiratory chain enzyme activity but not platelet function in this group of patients.

### Platelet mitochondrial biochemistry

Ethylene diamine tetra-acetic acid-anticoagulated venous blood was sedimented for 45 minutes at 4°C. The top three-quarters of platelet-rich plasma were centrifuged at 5,000 × *g* for 10 minutes. The pellet was washed with distilled water, centrifuged at 14,500 × *g* for 10 minutes, washed again with phosphate-buffered saline and finally stored at −80°C. At the time of analysis, the platelet pellet was diluted in buffer (KCl 120 mM, 4-(2-hydroxyethyl)-1-piperazine ethanesulfonic acid 20 mM, MgCl_2_ 5 mM and ethylene glycol tetra-acetic acid 1 mM; pH 7.2, 300 to 400 μl), sonicated (two cycles at 60 W for 10 seconds) and centrifuged (750 × *g* for 10 minutes) while kept at 4°C. Supernatant was then analysed using spectrophotometry at 30°C. We measured the activity of nicotinamide adenine dinucleotide dehydrogenase (NADH), NADH–ubiquinone 1 reductase (complex I), NADH–cytochrome *c* reductase (complex I + III), succinate dehydrogenase, succinate dehydrogenase–cytochrome *c* reductase (complex II + III) and cytochrome *c* oxidase (complex IV), the main components of the mitochondrial respiratory chain [4], and expressed it relative to citrate synthase activity, a marker of mitochondrial density [5-7]. Proteins were measured with Lowry’s method. Data referred to seven patients with sepsis herein reported have also been presented in another publication [7].

### Platelet aggregation and secretion

Platelet aggregation and secretion were recorded on a Chrono-Log lumiaggregometer (Mascia Brunelli, Milan, Italy). Briefly, 3.8% citrate-anticoagulated venous blood was centrifuged (1900 × *g* for 15 minutes) to obtain platelet-rich plasma. After separation of platelet-rich plasma, tubes were centrifuged again (1,100 × *g* for 15 minutes) to obtain platelet-poor plasma. Platelet-rich plasma (450 μl) was mixed with luciferase reagent (50 μl) and stirred at 1,000 rpm at 37°C. After 30 seconds, one of the following agonists that activate different signal transduction pathways was added (final concentration): ADP (Sigma Aldrich, St. Louis, MO, USA) 4 μmol/l, collagen (Mascia Brunelli) 2 μg/ml, thromboxane A_2_ analogue U46619 (Sigma Aldrich) 0.5 μmol/l, and thrombin receptor activating peptide (Sigma Aldrich) 10 μmol/l. At this dose, ADP elicits a biphasic platelet response: the first wave of aggregation in response to exogenous ADP is followed by δ-granule secretion and the second wave of aggregation in response to released endogenous adenine nucleotides. By contrast, with other agonists, aggregation and secretion occur at the same time, so that the first wave cannot be distinguished from the second wave of aggregation [21]. First wave (with ADP) and maximal (over 3 minutes; with all agonists) aggregation was measured as the decrease in optical density of platelet-rich plasma, considering platelet-poor plasma (lowest optical density) as the reference (100% aggregation). Platelet secretion was assessed as the release of ATP.

### Platelet nucleotides

Total ATP and ADP contents were measured with a LKB 1250 luminometer (Bio-Orbit Oy, Turku, Finland) using the firefly luciferin–luciferase method (Promega Corporation, Madison WI, USA). Platelet serotonin content was measured with the *o*-phthaldialdehyde system [22].

### Plasma interleukin-6

Interleukin-6 was measured in duplicate with enzyme-linked immunosorbent assays (RayBiotech, Norcross, GA, USA).

### Statistical analysis

Data are reported as mean (± standard deviation) or median (interquartile range). Groups were compared using Student’s *t* test or the Wilcoxon rank-sum test, one-way analysis of variance or one-way analysis of variance on ranks. Proportions were compared with the chi-square test or Fisher’s exact test. The strength of association between variables was measured with linear regression analysis or Spearman’s rank correlation. *P* <0.05 indicated statistical significance (SigmaPlot; Jandel Scientific Software, San Jose, CA, USA).

## Results

The main characteristics of subjects enrolled in the study are presented in Table 1. Patients with severe sepsis (*n* = 3) or septic shock (*n* = 13) had pulmonary (*n* = 9), abdominal (*n* = 6) or skin (*n* = 1) infection. Those with cardiogenic shock had acute myocardial infarction (*n* = 10), pulmonary embolism (*n* = 2), malignant arrhythmia (*n* = 2), pericardial tamponade (*n* = 1) or prosthetic mitral valve failure (*n* = 1) eventually complicated by transient cardiac arrest (*n* = 10).

**Table 1:** **Main characteristics of subjects enrolled in the study**

	**Healthy controls**	**Sepsis**	**Cardiogenic shock**	***P*** **value**
*n*	16	16	16	
Sex (male/female)	7/9	7/9	9/7	0.716
Age (years)	61 ± 11	62 ± 16	66 ± 17	0.306
Diabetes	1	2	6	0.110
Obesity	1	4	5	0.248
Smoking history	3	3	3	1.000
SAPS II	–	42 ± 12	75 ± 17	<0.001
SOFA score	–	9 (6 to 11)	12 (11 to 14)	<0.001
Respiration	–	3 (2 to 4)	3 (2 to 4)	0.984
Coagulation	–	1 (0 to 2)	0 (0 to 0)	0.007
Liver	–	0 (0 to 1)	0 (0 to 1)	0.748
Cardiovascular	–	4 (3 to 4)	4 (3 to 4)	0.746
Central nervous system	–	0 (0 to 0)	4 (3 to 4)	<0.001
Renal	–	0 (0 to 1)	1 (1 to 2)	0.022
Platelet count (× 10^3^/mm^3^)	229 (191 to 268)	140* (93 to 183)	180 (153 to 221)	<0.001
Central (or mixed) venous oxygen saturation (%)	–	74 ± 9	62 ± 14	0.015
Blood lactate (mmol/l)	–	2 ± 2	10 ± 5	<0.001
Antibiotic(s)	–	16	6	<0.001
Sedative(s) intravenously	–	13	12	1.000
Catecholamine(s)	–	13	16	0.226
Mechanical ventilation	–	15	15	1.000
Renal replacement therapy	–	0	1	1.000
Intra-aortic balloon pump	–	0	2	0.484
Days from hospital to ICU admission	–	0 (0 to 1)	1 (0 to 8)	0.074
Hours from ICU admission to study enrolment	–	22 ± 13	9 ± 6	<0.001
Length of stay in ICU (days)	–	16 (6 to 24)	1 (1 to 2)	<0.001
Deaths in ICU	–	5	10	0.156

Data reported as number, mean ± standard deviation or median (interquartile range). Patients with sepsis or cardiogenic shock entered the study within 48 hours from admission to intensive care (ICU). Obesity was defined as body mass index ≥30 kg/m^2^. Smoking history was not known for two patients with sepsis and two patients with cardiogenic shock. Simplified Acute Physiology Score (SAPS) II and Sequential Organ Failure Assessment (SOFA) scores refer to the first day in the ICU. Central (*n* = 16) or mixed (*n* = 12) venous oxymetry (four missing values) and blood lactate levels (one missing value) are the worst values recorded from ICU admission to study enrolment. Use of artificial organ support refers to this same period of time. *P* values refer to Student’s *t* test or the Wilcoxon rank-sum test, one-way analysis of variance (ANOVA) or one-way ANOVA on ranks (*P <0.05 vs. healthy controls on *post hoc* all-pairwise multiple comparisons (Tukey or Dunn’s test)), chi-square test or Fisher’s exact test. In the case of two row × three column contingency tables, the Freeman–Halton extension of Fisher’s exact test was used as appropriate.

Patients with sepsis usually received carbapenems, glycopeptides, broad-spectrum penicillins and/or quinolones, benzodiazepines and opioids, norepinephrine and/or dopamine. Those with cardiogenic shock were occasionally treated with clindamycin or broad-spectrum penicillin (in case of aspiration) and always received benzodiazepines or propofol, opioids and catecholamine(s) (mainly dopamine and/or epinephrine). Thirteen patients with cardiogenic shock received salicylic acid or its derivatives.

### Platelet mitochondrial biochemistry

**Figure 1:**
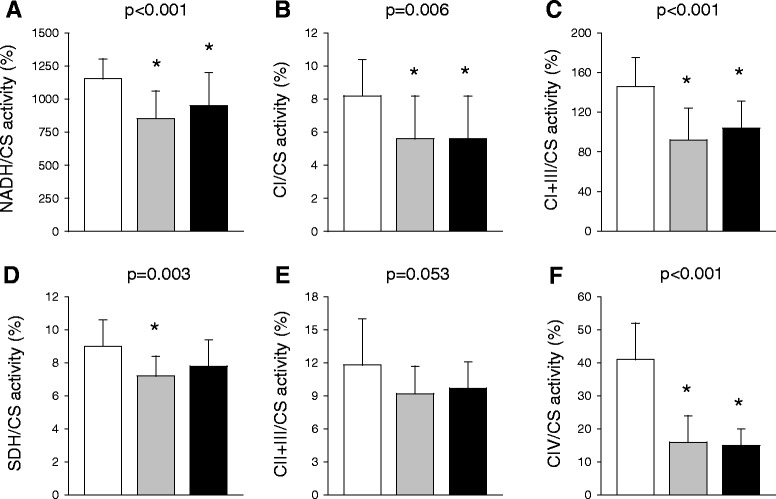
**Platelet mitochondrial biochemistry during sepsis or cardiogenic shock.** Activity of platelet respiratory chain enzymes was measured in healthy volunteers (white bars) and patients with sepsis (grey bars) or cardiogenic shock (black bars) (within 48 hours from admission to intensive care). Nicotinamide adenine dinucleotide dehydrogenase (NADH) **(A)**, complex I (CI) **(B)**, complex I + III (CI + III) **(C)**, succinate dehydrogenase (SDH) **(D)**, complex II + III (CII + III) **(E)**, and complex IV (CIV) **(F)** activity is expressed relative to citrate synthase (CS) activity, a marker of mitochondrial density. *P* values refer to one-way analysis of variance (ANOVA) or one-way ANOVA on ranks (*P <0.05 vs. healthy volunteers on *post hoc* all-pairwise multiple comparisons (Tukey or Dunn’s test)). CS activity was 68 ± 8 nmol/minute/mg proteins in healthy volunteers, 67 ± 12 nmol/minute/mg proteins in patients with sepsis and 59 ± 9 nmol/minute/mg proteins in those with cardiogenic shock (*) (*P* = 0.026). Complex I, NADH–ubiquinone 1 reductase; complex I + III, NADH–cytochrome *c* reductase; complex II + III, succinate dehydrogenase–cytochrome *c* reductase; complex IV, cytochrome *c* oxidase.

Platelets of patients with sepsis or cardiogenic shock similarly had lower NADH (20 to 25% reduction), complex I (30% reduction), complex I and III (30 to 35% reduction) and complex IV (60 to 65% reduction) activity than those of healthy volunteers. Findings were similar in patients with cardiogenic shock treated with or without salicylic acid or its derivatives (*P* ≥0.414 for the activity of all respiratory chain enzymes). Platelets of patients with sepsis also had lower succinate dehydrogenase activity (20% reduction) than those of controls (Figure 1).

### Platelet aggregation and secretion

As specified above, platelet function was not assessed in patients with cardiogenic shock (*de facto*, only one of these patients had not received antiplatelet drugs by the time of enrolment).

**Figure 2:**
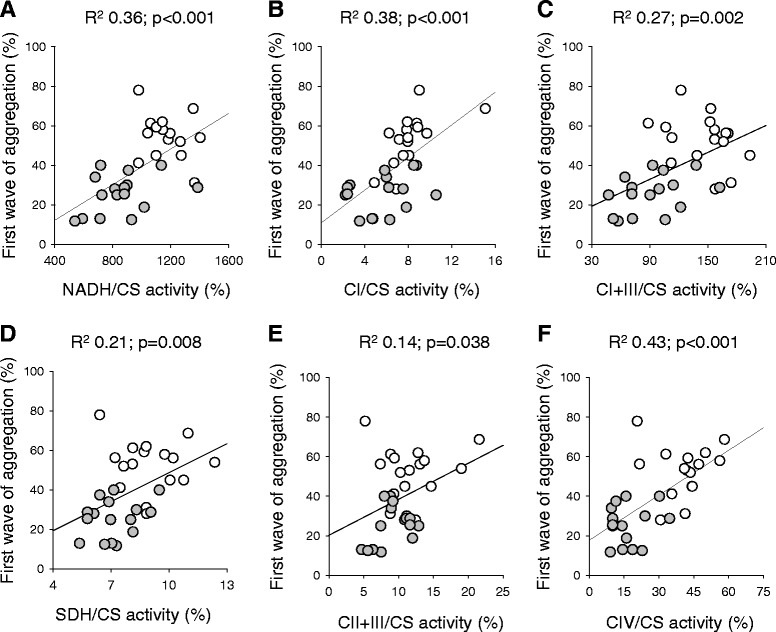
**Relationship between platelet mitochondrial biochemistry and the first wave of aggregation.** Activity of platelet respiratory chain enzymes and the first wave of aggregation with ADP (4 μmol/l) were measured in healthy volunteers (white dots) and in patients with sepsis (grey dots) (within 48 hours from admission to intensive care). Nicotinamide adenine dinucleotide dehydrogenase (NADH) **(A)**, complex I (CI) **(B)**, complex I + III (CI + III) **(C)**, succinate dehydrogenase (SDH) **(D)**, complex II + III (CII + III) **(E)**, and complex IV (CIV) **(F)** activity is expressed relative to citrate synthase (CS) activity, a marker of mitochondrial density. Aggregation was expressed as a percentage, where optical density of unstimulated platelet-rich plasma represents 0% and that of platelet-poor plasma is 100%. Strength of association between (normally distributed) variables was assessed with linear regression analysis and expressed as *R* ^2^. CS activity (expressed as nmol/minute/mg proteins) was not associated with the first wave of aggregation with ADP (*R* ^2^ = 0.00; *P* = 0.703). Complex I, NADH–ubiquinone 1 reductase; complex I + III, NADH–cytochrome *c* reductase; complex II + III, succinate dehydrogenase–cytochrome *c* reductase; complex IV, cytochrome *c* oxidase.

In patients with sepsis, the platelet first wave of aggregation (in response to ADP), maximal aggregation and secretion of ATP (in response to ADP, collagen, U46619 and thrombin receptor activating peptide) were significantly lower than those of controls (Table 2). The first wave of aggregation generally correlated with the activity of several platelet mitochondrial respiratory chain enzymes (Figure 2).

**Table 2:** **Platelet response to exogenous agonists during sepsis**

	**Healthy controls**	**Sepsis**	***P*** **value**
*n*	16	16	
Maximal aggregation (%)			
With ADP (4 μmol/l)	65 ± 18	30 ± 14	<0.001
With collagen (2 μg/ml)	80 ± 9	36 ± 17	<0.001
With U46619 (0.5 μmol/l)	75 ± 19	31 ± 18	<0.001
With thrombin receptor activating peptide (10 μmol/l)	72 ± 27	21 ± 17	<0.001
First wave of aggregation (%)			
With ADP (4 μmol/l)	53 ± 13	26 ± 10	<0.001
Secretion (nmol ATP/10^8^ platelets)			
With ADP (4 μmol/l)	0.20 ± 0.18	0.07 ± 0.10	0.041
With collagen (2 μg/ml)	0.67 ± 0.23	0.35 ± 0.37	0.002
With U46619 (0.5 μmol/l)	0.28 ± 0.13	0.12 ± 0.13	0.003
With thrombin receptor activating peptide (10 μmol/l)	0.45 ± 0.26	0.13 ± 0.21	<0.001

Data reported as mean ± standard deviation. Platelet maximal aggregation and secretion (in response to ADP, collagen, thromboxane A_2_ analogue U46619 and thrombin receptor activating peptide) and first wave of aggregation (in response to ADP) were measured in healthy volunteers and patients with sepsis (within 48 hours from admission to intensive care). Aggregation is expressed as a percentage, where optical density of unstimulated platelet-rich plasma represents 0% and that of platelet-poor plasma is 100%. Secretion was measured as ATP released in the extracellular space (nmol/10^8^ platelets). A curve obtained by adding a fixed dose of ATP to platelet-poor plasma was used as standard. Please note that the platelet count in platelet-rich plasma of patients was above 100 × 10^3^ platelets/mm^3^ in all but two cases. Blood fibrinogen levels in patients with sepsis were 522 ± 202 mg/ml (internal reference values: 200 to 400 mg/ml). *P* values refer to Student’s *t* test or the Wilcoxon rank-sum test.

### Platelet nucleotides

Platelets of patients with sepsis had a lower level of total ADP (1.4 ± 1.0 vs*.* 2.6 ± 0.7 nmol/10^8^ platelets; *P* <0.001), a similar level of total ATP (6.0 ± 2.0 vs*.* 5.9 ± 1.6 nmol/10^8^ platelets; *P* = 0.910) and a higher ATP-to-ADP ratio (6.8 ± 5.3 vs*.* 2.3 ± 0.5; *P* < 0.001) than those of healthy volunteers. They also contained less serotonin than those of controls (0.29 ± 0.20 vs*.* 0.48 ± 0.15 nmol/10^8^ platelets; *P* = 0.006).

### Plasma interleukin-6

Interleukin-6 was higher in patients with sepsis (187 (86 to 615) pg/ml; *n* = 15; *P* <0.05) or cardiogenic shock (387 (43 to 626) pg/ml; n = 15; *P* <0.05) than in controls (4 (1 to 15) pg/ml; *n* = 10).

## Discussion

This study demonstrates that platelet mitochondrial respiratory chain enzymes are similarly inhibited in patients with sepsis or cardiogenic shock and that these changes, during sepsis, are associated with general platelet hyporesponsiveness.

Platelet mitochondrial dysfunction is not specific to sepsis. Mitochondrial changes occurring in blood cells during severe infection are commonly attributed to circulating factors [23-26], possibly including inflammatory mediators and drugs. However, systemic inflammation can also develop in response to sterile insults [27,28] and high levels of cytokines can be found in blood even after cardiac failure [29], as confirmed by our own data on interleukin-6. Patients with cardiogenic shock as well as those with sepsis commonly received antibiotics, sedatives or catecholamines that are potentially toxic for the mitochondrion [30,31]. Accordingly, plasma of patients resuscitated from cardiac arrest can cause mitochondrial dysfunction *in vitro* [32]. These results, however, do not exclude a major role for infection (compared with hypoperfusion) in the development of mitochondrial dysfunction. In fact, respiratory chain enzymes were always similarly inhibited although the severity of disease (Table 1) and inflammatory response (circulating interleukin-6 levels) were apparently lower during sepsis than during cardiogenic shock.

Patients with cardiogenic shock, but not those with sepsis (see inclusion criteria), were almost always treated with salicylic acid or its derivatives. According to some authors, these compounds may directly interfere with (platelet) mitochondrial function, mainly acting as uncoupling agents [33]. However, in our study population, severe platelet mitochondrial inhibition (rather than uncoupling) was equally observed in subjects treated with or without salicylic acid or its derivatives. Preliminary experiments in our laboratory confirm that complex IV activity (that was largely depressed in patients with cardiogenic shock) remains normal in healthy volunteers treated with aspirin (100 mg/day) for 3 days. Based on these findings, salicylic acid and its derivatives probably had only a minor (if any) role in the development of platelet mitochondrial dysfunction during cardiogenic shock.

Along with signs of mitochondrial dysfunction, platelets of patients with sepsis were hyporesponsive to several agonists (as already shown by other authors [34-37]), had abnormally low intracellular levels of ADP and serotonin (main granular components) and had an abnormally high ATP-to-ADP ratio (that approximated cytoplasmic values). Total ATP levels that mainly depend on the cytoplasmic pool were grossly preserved. Overall, these changes suggest that secretion had already occurred *in vivo* leaving platelets functionally exhausted, with empty δ-granules, unable to further respond to agonists (acquired δ storage pool disease) [38,39]. Maximal aggregation diminished in line with secretion and granular content, as it does during inherited defects of δ-granules [40,41]. This model, however, does not account for observed inhibition of the first wave of aggregation in patients with sepsis. In fact, the first wave of aggregation precedes secretion and is usually normal even in patients with δ storage pool disease [40,41]. Other mechanisms should then be considered, probably including incapacity of platelets to accelerate their energy turnover because of inhibition of key mitochondrial enzymes [42]. Sodium azide, a well-known blocker of mitochondrial respiration, similarly interferes with first wave aggregation induced by ADP in human platelets [43].

Platelet hyporesponsiveness during sepsis may thus represent, at least in part, an adaptive response to acquired platelet mitochondrial dysfunction, with the aim of preserving a physiological balance between energy demand and provision. Large inhibition of complex IV probably has a crucial role in this sequence of events, both limiting cellular respiration and triggering a decrease in ATP consumption [44].

Some limitations of our study deserve comment. The sample size was small. Enrolment of patients with sepsis was particularly difficult due to common use of nonsteroidal anti-inflammatory drugs (exclusion criteria). Changes occurring in platelets may not reflect those occurring in other tissues or organs [7]. In addition, spectrophotometry and lumiaggregometry evaluate *ex vivo* properties of circulating platelets. Therefore, they may only partly describe changes that occur *in vivo* in platelets eventually entrapped in the microcirculation. Other aspects of platelet mitochondrial physiology, such as oxygen consumption or membrane potential, were not investigated. Platelet mitochondrial density was evaluated in terms of citrate synthase activity (normal even in critically ill patients) and not with more sophisticated techniques, including electron microscopy. Some authors have noted that platelet citrate synthase activity relates to other markers of mitochondrial content, such as mitochondrial DNA, and remains normal during human sepsis [26]. However, one recent study has demonstrated that activated platelets release some of their mitochondria in the extracellular milieu with electron microscopy [45]. Platelet citrate synthase activity may thus be normal in face of some change in mitochondrial density. Loss of organelles is expected to result in a parallel decrease in the activity of all mitochondrial enzymes. By contrast, we have noted a selective, large inhibition of some enzymes but not of others in critically ill patients, as if platelet mitochondria had a defect in their activity and not just in their density. Finally, whether mitochondrial dysfunction was responsible for, and not only associated with, platelet hyporesponsiveness remains unclear. For all these reasons, our results should be interpreted with caution.

## Conclusions

Several platelet mitochondrial respiratory chain enzymes are inhibited during human sepsis or cardiogenic shock. In patients with sepsis, these mitochondrial changes are related to general platelet hyporesponsiveness to exogenous stimulation.

## Key messages

- Platelet mitochondrial respiratory chain enzymes are similarly inhibited in patients with sepsis or cardiogenic shock.
- In patients with sepsis, platelet mitochondrial changes are related to general platelet hyporesponsiveness to exogenous agonists.
- In patients with sepsis, circulating platelets also suffer from acquired δ storage pool disease.

## Abbreviations

δ: dense
NADH: nicotinamide adenine dinucleotide dehydrogenase
